# Anomalous constitutive Src kinase activity promotes B lymphoma survival and growth

**DOI:** 10.1186/1476-4598-8-132

**Published:** 2009-12-31

**Authors:** Jiyuan Ke, R Lakshman Chelvarajan, Vishal Sindhava, Darrell A Robertson, Lazaros Lekakis, C Darrell Jennings, Subbarao Bondada

**Affiliations:** 1 Department of Microbiology, Immunology & Molecular Genetics, University of Kentucky, Lexington KY 40536, USA; 2 Sanders Brown Center on Aging, University of Kentucky, Lexington KY 40536, USA; 3 Markey Cancer Center, University of Kentucky, Lexington KY 40536, USA; 4 Department of Internal Medicine, University of Kentucky, Lexington KY 40536, USA; 5 Department of Pathology & Laboratory Medicine, University of Kentucky, Lexington KY 40536, USA; 6 Van Andel Research Institute, Grand Rapids MI 49503, USA

## Abstract

**Background:**

Previously we have shown that B cell receptor (BCR) expression and B cell receptor signaling pathways are important for the basal growth of B lymphoma cells. In particular we have shown that the activation of Syk, a non-src family protein tyrosine kinase and the mitogen activated protein kinases (MAPK), ERK and JNK that mediate BCR signals are required for the constitutive growth of B lymphoma cells. Since src family protein tyrosine kinases (SFKs) like Lyn are known to be needed for the phosphorylation of BCR co-receptors, Ig-α and Ig-β, we hypothesized that one or more SFKs will be constitutively activated in B lymphoma cells and may be necessary for B lymphoma growth.

**Results:**

Src kinase activity was found to be constitutively high in many murine and human B lymphoma cell lines and primary lymphoma samples. The specific pharmacological inhibitors of SFKs, PP1 and PP2 inhibited the proliferation of a number of both murine and human B lymphomas in a dose-dependent manner. Importantly, dasatinib (BMS-354825), an oral dual BCR-ABL and SFK specific inhibitor inhibited the growth of B lymphomas in the nanomolar range in vitro and strongly inhibited a mouse lymphoma growth in vivo. Among the SFKs, Lyn is predominantly phosphorylated and Lyn-specific small interfering RNA inhibited the growth of B lymphomas, supporting an important role for Lyn in B lymphoma growth. Suppression of SFK activity blocks BCR mediated signaling pathways. PMA or CpG can partially reverse the growth inhibition induced by SFK inhibition. Although blocking SFK activity inhibited the growth of a number of B lymphomas, some lymphomas such as SudHL-4, SudHL-6, OCI-Ly3 and OCI-Ly10 are more resistant due to an increased expression of the anti-apoptotic proteins Bcl-2 and Bcl-x_L_.

**Conclusions:**

These studies further support our concept that BCR signaling pathways are important for the continued growth of established B lymphoma cells. Some of the intermediates in this BCR pathway are potential immunotherapeutic targets. In particular, inhibition of SFK activity alone or in synergy with inhibition of the prosurvival Bcl-2 proteins holds promise in developing more effective treatments for B lymphoma patients.

## Introduction

Non-Hodgkin lymphoma (NHL) is the fifth most common cancer in the United States. About 90% NHLs are of B-cell origin. Diffuse large B-cell lymphoma (DLBCL) (30-40%) and follicular lymphoma (20%) are the two most common NHLs [1]. The genetic hallmarks of B-cell lymphomas are reciprocal chromosomal translocations involving one of the Ig loci and a proto-oncogene, such as BCL2, BCL6 or c-Myc [1,2]. In addition to these translocation events, B lymphoma cells maintain dependence on B cell surface receptor (BCR) signaling for survival and growth [3].

B-cell lymphomas arise during various stages of B cell development. B-cell precursors in bone marrow differentiate into mature naïve B cells and leave the bone marrow only after a B-cell precursor successfully rearranges Ig H and L chains and expresses a functional BCR. During development, B cells undergo stringent selection for expression of the appropriate BCR. Expression of BCR is even required for the survival of mature resting B cells because ablation of BCR expression in mice leads to apoptosis of BCR-negative B cells [4]. B-cell lymphomas appear to be also under selective pressure to express BCR [1]. First, most B-cell lymphomas still express surface BCR. Second, translocations into the Ig-loci are virtually always found on the non-productively rearranged Ig loci. Third, treatment of patients who had follicular lymphoma with anti-idiotypic antibodies did not result in the emergence of BCR-negative lymphoma variants. Fourth, gene expression analysis demonstrated that BCR signaling pathways are elevated in a number of DLBCL that don't respond well to chemotherapy [5,6]. Finally, the siRNAs targeting Igα and Igβ caused suppression of B lymphoma growth [3]. These data suggested that the BCR complex provides survival signals for B lymphoma cells. Moreover, it was shown that proteins containing immunoreceptor tyrosine-based activation motifs (ITAM) are sufficient to cause transformation. A recombinant protein consisting of ITAM containing cytoplasmic regions of Igα and Igβ of BCR complex caused transformation of mammary epithelial cells and fibroblasts [7]. The Kaposi sarcoma-associated herpes virus K1 protein bearing ITAM motif induced plasmablastic lymphomas in K1 transgenic mice [8]. The ITAM containing proteins induced transformation presumably by acting as a scaffold for downstream mediators.

For B cell activation, BCR engagement by antigen leads to activation of Src kinase Lyn, which phosphorylates the ITAM motifs of Igα and Igβ of the BCR complex. The phosphorylated ITAM motifs recruit the Syk kinase to mediate multiple downstream signals to instruct normal B cells to make crucial cell-fate decisions in cell differentiation [9,10]. Since Lyn is also responsible for phosphorylating several inhibitory (ITIM-containing) receptors in B cells and myeloid cells, it was discovered to have a dual role acting both as a positive and a negative signaling molecule [11]. However, due to the ability of other SFKs to substitute for Lyn activity in B cells, BCR signaling is not interrupted in the complete absence of Lyn [12-15]. For T cell activation, the counterpart of Lyn is Src kinase Lck, which phosphorylates the ITAM motifs of CD3 of the TCR complex. In both cases, Src kinases are essential for receptor mediated early signaling events required for B (or T) cell survival and activation. Syk has been found to be constitutively active in B lymphomas and inhibitors of Syk reduce growth of B lymphoma cells [16,17].

SFKs are non-receptor protein tyrosine kinases with nine known members, Src, Yes, Fyn, Lyn, Lck, Hck, Fgr, Blk and Yrk. In addition to their role in mediating immune response as mentioned above for Lyn and Lck, SFKs are also involved in the control of cellular processes such as cell survival, proliferation, differentiation, phagocytosis, angiogenesis, adhesion, motility [11,18-22]. Each SFK has a unique N-terminal domain followed by three conserved Src homology domains: SH3, SH2 and SH1. All SFKs are myristoylated at the N terminus, which targets them to the cell membrane. They are regulated by phosphorylation at two critical tyrosines with opposing effects. Phosphorylation at the C-terminal tyrosine (Y527 for c-Src) by C-terminal kinase (Csk) suppresses its activity whereas phosphorylation of the tyrosine (Y416 for c-Src) in the activation loop of the kinase domain (SH1) up regulates its activity.

c-Src, the archetypal member of SFKs, is implicated in a large number of human cancers including colorectal, hepatocellular, pancreatic, breast, ovarian and lung cancers [18,23]. Blk is preferentially expressed in B cell lineage and involved in the early development of B cells. Expression of constitutively active Src kinase Blk (Y495F) in B and T lymphoid compartment induces transformation of specific B and T cell progenitor cells into lymphomas [24,25]. Studies show that Src kinase Lyn is the predominant cellular Src activity in glioblastoma tumor cells [26] and chronic lymphocytic leukemia B cells [27] and promotes the malignant phenotype in these tumors. Lyn also plays an important role for chronic myelogenous leukemia (CML) blast crisis cells and Lyn siRNA induces apoptosis of drug (Gleevec) resistant BCR-ABL1(+) cells [28]. In another study, at least two SFKs were required for efficient induction of B-lymphoid leukemia by BCR-Abl [29].

Together with the data on the importance of SFKs in leukemias and our finding that BCR signaling is required for basal B lymphoma growth, we hypothesized that Src kinase activity, especially Lyn activity, is elevated in B lymphoma cells and that the elevated Src kinase activity promotes B lymphoma growth. Despite some studies with cell lines [30], there is little information about Lyn activation in primary lymphoma cells, its role in BCR dependent lymphoma growth, and its importance for *in vivo *B lymphoma growth. Consistent with this hypothesis, we observed constitutively active Src kinase activity in a number of primary B lymphoma cells and lymphoma cell lines but not in normal B cells. DLBCLs were used to evaluate the importance of SFK for B lymphoma growth. Specific pharmacological inhibitors of SFK (PP1, PP2 and dasatinib) induced a dose dependent inhibition of B lymphoma cell growth due to G_1_-S arrest. dasatinib strongly inhibited the BKS-2 lymphoma growth *in vivo *in a mouse lymphoma model. Although other members of SFK were expressed variably in lymphoma cells, Lyn is the predominant kinase that is constitutively phosphorylated and appears to be critical for B lymphoma growth. We demonstrated that inhibition of SFK reduced BCR signaling.

## Materials and methods

### Reagents

PP1, PP2, and PP3 were obtained from BIOMOL International, L.P. (Plymouth Meeting, PA). dasatinib was obtained from the University of Kentucky Hospital. Phospho-specific antibodies against Src(Tyr416), Lyn(Tyr507), JNKs(Thr183/Tyr185), CD19(Tyr513), ERKs(Thr202/Tyr204) and AKT(Ser473), phospho-Tyrosine (P-Tyr100, biotinylated) were obtained from Cell Signaling Technologies (Beverly, MA). Antibodies against total Src, Fgr, Fyn, Hck, Yes, AKT were also obtained from Cell Signaling Technologies (Beverly, MA). Antibodies against total Lck, Lyn, Blk, Igα, cyclin D2, Egr-1(C-19), ERK1, Bcl-XL and Bcl-2 were obtained from Santa Cruz Biotechnology, Inc. (Santa Cruz, CA). Monoclonal anti-β-actin antibody was obtained from Sigma-Aldrich (St. Louis, MO). The siRNA against human Lyn and control siRNA were obtained from Dharmacon, Inc (Lafayette, CO). The phosphorothioate-modified CpG oligonucleotides (ODN) 3Db as described by Krieg et al. [31] was obtained from the Regional DNA Synthesis Laboratory (Calgary, Canada).

### Cells and Mice

Female CBA/N (Xid) mice were purchased from The Jackson Laboratory (Bar Harbor, ME). Mice were housed under specific pathogen-free conditions in micro-isolator cages under the American Association for Laboratory Animal Accreditation and Certification approved protocol. B lymphoma cell lines of both murine and human origins were described previously [3]. Primary human B lymphoma cells were obtained from anonymized discarded flow cytometry samples under an IRB exemption protocol. Human peripheral blood lymphocytes (PBLs) were obtained from discarded samples generated by the Central Kentucky Blood Center during RBC enrichment. Mononuclear cells were obtained after subjecting PBLs to centrifugation on a Ficoll-Hypaque cushion [32] and then B cells were enriched with CD19 microbeads (Miltenyi Bergisch Gladbach, Germany) using the manufacturer's protocol. The isolation and characterization of BKS-2 and host T cell depletion have been described previously [33,34]. BKS-2 cells were grown in female CBA/N (Xid) mice as splenic tumors by intravenous injection. These cells attained maximal growth (2-6 × 10^8 ^cells per mouse spleen) in 7-10 days and were collected for experimental use at this stage.

### Western Blot Analysis and Immunoprecipitation

Various B lymphoma cells (5 × 10^6^) with or without treatments were cultured at 1 × 10^6^/ml in 6-well plates (Costar) for the indicated time. Cell pellets were lysed in a buffer with 1% Triton X-100 and protease inhibitors and processed for Western blots as described [35-37]. The blots were developed with Pico chemiluminescence substrate (Pierce) and exposed to Kodak X-Omat films, or analyzed by an Eastman Kodak Image Station 2000RT. For re-probing, membranes were stripped using a solution containing 62.5 mM Tris-HCl, 2% SDS, and 100 mM β-mercaptoethanol at 62°C for 10 min. For immunoprecipitation, the cell lysates were pre-cleared by incubation with 50 μl protein G beads (50% v/v) at 4°C for 1 hr. The cleared lysate was incubated with 2-5 μg of antibody for 2 hrs at 4°C. The immune complex was isolated on protein G beads (50% v/v) and was analyzed by Western blot. Densitometry for bands on Western blots was quantified using the Gel Analysis method of the ImageJ program according to its documentation http://rsb.info.nih.gov/ij/[38].

### Transfection of siRNA into B Lymphoma Cells

The sequence of Lyn-specific siRNA used in this study was obtained from a successful previous attempt to repress Lyn protein. The sense and antisense sequences of human Lyn-specific siRNA were 5'-AGACUCAACCAGUACGUAAUU-3' and 5'-PUUACGUACUGGUUGAGUCUUU-3', respectively [28]. The non-specific control siRNA with Cat# D-001206-01-20 was used. Lyn specific siRNA or the control siRNA was introduced into B lymphoma cells by electroporation. 5-8 × 10^6 ^lymphoma cells were washed, resuspended in cold Opti-MEM I reduced serum media (GIBCO) mixed with 500 nM of control or Lyn specific siRNA and electroporated at 260 mV, 960 microfarads, and 200 ohms. The transfection efficiency for SudHL-4 and -6 cell lines was determined to be about 70%, based on co-transfection with a GFP expressing plasmid. One day post-electroporation, lymphoma cells were counted, and an equal number of cells with the indicated treatment were used to set up the proliferation assay as described.

### Cell Proliferation and Cell Cycle Assays

Lymphoma cells (2-3 × 10^4^/well) were cultured in 96-well flat-bottom microtiter plates (Costar, Cambridge, MA) in 200 μl of media with 10% FCS. The cells were pulsed with 1 μCi of [^3^H] thymidine (PerkinElmer Life Sciences) during the last 4 hrs of the 48 hrs culture period. The cells were harvested and the radionucleotide incorporation was measured with a Matrix 96 β-radiation counter (Hewlett-Packard, Downers Grove, IL). Results are presented as the means ± S.E. of triplicate cultures. The percent control response is defined as (cpm in the treated group/cpm in the untreated group) × 100. To determine the IC_50 _a linear regression was plotted between points near 50% inhibition and the resulting equation was used to determine the dose that caused 50% growth inhibition.

The cell cycle was analyzed using propidium iodide (PI). B lymphoma cells (0.5 × 10^6^/ml) were treated with varying doses of PP1 or PP2 and then fixed in 70% (v/v) ethanol for at least 1 h at 4°C, after which cells were incubated in a mixture of 1 μg/ml PI (Sigma-Aldrich) and 25 μg/ml RNase A (Sigma-Aldrich) at 37°C for 30 min. The level of PI fluorescence was measured with a MoFlo flow cytometer (DakoCytomation). Cell populations at subG_1_, G_1_, S, G_2_/M phase were calculated using the program ModFit.

### Cell Apoptosis Analysis

B lymphoma cells were treated with various doses of inhibitors for one to three days and stained with Annexin V (FITC or APC) (BD Pharmingen) at room temperature for 15 min in the dark. Then 3 μl of PI solution (0.5 μg/ml) was added and samples were analyzed by flow cytometry within one hour.

### *In vivo *tumor inhibition study

2 month old female CBA/N (Xid) mice were injected intravenously with 7 × 10^6 ^BKS-2 B lymphoma cells on day 0. From day 1, mice were injected intraperitoneally either with 1 mg/kg body weight dasatinib in 1 × PBS with 10% DMSO or 200 μl of vehicle (1 × PBS, 10% DMSO) everyday for 14 days. Mice were sacrificed afterwards and spleens were removed to count for total number of splenic tumor cells.

## Results

### SFKs are constitutively activated in a number of primary B lymphomas and B lymphoma cell lines

**Figure 1:**
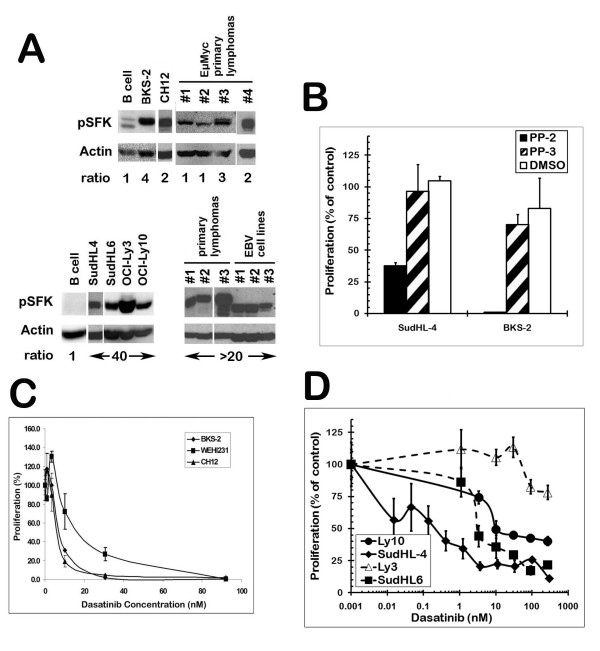
**B lymphomas have high SFK activity as compared to normal B cells**. (Panel A) The top row of blots is of mouse and the bottom row is of human B cells. SudHL-4 and 6, Ly3 and 10, and human primary lymphoma #1 are diffuse large B cell lymphomas while the murine primary lymphomas were from EμMyc transgenic mice. Human primary lymphomas #2 & 3 are small cell lymphomas. The cell lysates were first probed for phospho-SFK with p-Src (Y416) antibody, then stripped and re-probed for β-actin as a loading control. The ODs for the p-SFK bands were normalized to the corresponding β-actin bands. The ratios are provided under each lane and are depicted as a fold change over normal cells (i.e. murine lymphomas as a ratio to murine splenic B cells and human lymphomas as a ratio to PB B cells). (B) Cultures of murine (BKS-2) and human (SudHL-4) B lymphomas were treated with the SFK inhibitor PP2 (10 μM) or with the inactive control analog, PP3 (10 μM) for 48 hr and then the proliferation was measured by [^3^H]-thymidine uptake. Cultures of murine (C) and human (D) B lymphomas were treated with various doses of dasatinib (a dual BCR-ABL and SFK inhibitor) for 48 hr and then the proliferation was measured by [^3^H]-thymidine uptake. The proliferation assay was performed as described in the "Materials and Methods" section. Data points indicate percent control response ± S.E. of triplicate cultures from a representative experiment. The percent control response is defined as (cpm in the treated group/cpm in the untreated group) × 100. The levels of tritiated thymidine incorporation in the untreated groups were between 34,767 and 84,547 cpm.

Since SFKs play a key role in B-lymphoid transformation [39] we examined the levels of active SFKs present in B lymphoma lines, primary lymphoma tumor samples, and normal B cells (Fig. 1A). Phospho-Src (Y416) antibody specifically detects phosphorylation of tyrosine 416 at the activation loop of Src, an indication of active form of Src. It also cross-reacts with other Src family protein tyrosine kinases phosphorylated at equivalent position. Compared to normal murine splenic B cells, the level of active SFK was significantly elevated in murine lymphoma cell lines (BKS-2 and CH12) and two murine primary lymphomas from EμMyc transgenic mice (#3 and #4). The level of active SFK was also significantly elevated in DLBCL cell lines (SudHL6, OCI-Ly3 and OCI-Ly10), primary lymphoma samples, and EBV transformed B cells over normal human peripheral blood B cells (Fig. 1A).

### Blocking SFK activity suppresses B lymphoma cell growth

To block SFK activity, lymphoma cells were treated in vitro with the widely used synthetic pyrazolopyrimidine compounds, PP1 and PP2 or the inactive analogue, PP3 [40]. Treatment with PP2 potently inhibited the growth of BKS-2 and SudHL-4 cells while the inactive analog PP3 had no effect (Fig. 1B). PP2 also inhibited several other B lymphoma cell lines of human and murine origin (Table 1). A similar pattern was also observed for another SFK inhibitor, PP1 with slightly lower potency for some of the cell lines (Table 1). The IC_50 _of PP1 and PP2 for most cell lines were in the micromolar range. OCI-Ly3 had the highest IC_50 _for PP1 and PP2 compared with other cell lines, which correlated well with the highest level of phospho-SFK in the OCI-Ly3 cell line.

**Table 1:** The IC_50 _of SFK inhibitors PP1 and PP2 and BCR-Abl kinase/Src kinase inhibitor Dasatinib on a panel of B lymphoma cells of human and murine origin.

	**IC**_**50**_	PP1(μM)	PP2 (μM)	dasatinib (nM)
**Murine lymphoma**	BKS-2	8.0	7.4	8.3
	
	WEHI-231	4.6	2.8	17.5
	
	CH31	6.1	3.6	-
	
	CH12.Lx	2.5	2.8	7.3

**Human lymphoma**	SudHL-4	1.9	3.1	30
	
	SudHL-6	6.6	5.2	2.9
	
	OCI-Ly-3	19.5	12.6	1400
	
	OCI-Ly-10	3.3	2.0	9.9
	
	BJAB	4.6	3.4	-

The IC_50 _was determined using the equation derived from a linear regression of the data.

Dasatinib (BMS-354825) is an oral dual BCR/ABL and SFK inhibitor approved for use in patients with CML and Philadelphia chromosome-positive acute lymphoblastic leukemia [41-44]. Dasatinib very potently inhibited the growth of a number of B lymphoma cells. The IC_50 _of dasatinib for most cell lines was at nanomolar concentrations, one thousand fold less than the PP1 or PP2 inhibitors. In general, murine cell lines BKS-2, WEHI-231, CH12 were inhibited by lower doses of dasatinib than the human cell lines SudHL-6, OCI-Ly10 and SudHL-4 (Fig. 1C &1D). The OCI-Ly3 cell line required much higher dose of dasatinib to inhibit its growth, consistent with its highest level of phospho-SFK (Fig. 1A and Table 1). There was a small increase in growth of B lymphoma cells at low doses of dasatinib (Fig. 1C) and PP1 (data not shown). This could be due to a negative role for SFK in B lymphoma growth. Lyn is well documented to have both positive and negative roles in B cell proliferation and in myeloid cells [11,19]. The negative role of Lyn is in part due to its ability to phosphorylate tyrosine phosphatases, such as SHP-1 and SHIP-1 [45-47]. The enhancement seen at low doses of dasatinib may also relate to the ability of dasatinib to bind CSK, a negative regulator of SFK [48].

### Blocking SFK activity induces G1/S growth arrest accompanied by apoptosis of B lymphomas

**Figure 2:**
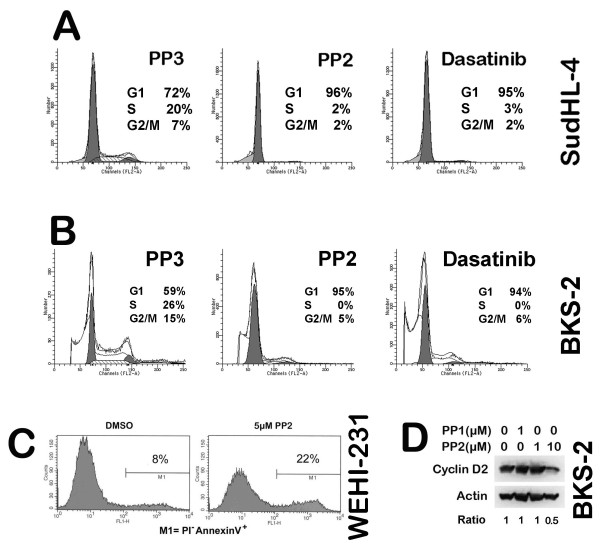
**PP2 and dasatinib induced G_1 _cell cycle arrest, accompanied by apoptosis in B lymphoma cells**. Cultures of 0.5 × 10^6^/ml human SudHL-4 cells (Panel A) or murine BKS-2 (B) were treated with 10 μM PP2 (SFK inhibitor), PP3 (inactive control analog), or 100 nM dasatinib (dual BCR-ABL and SFK inhibitor) for 48 hr. Then the cells were spun down and stained with PI for cell cycle analysis as described in the Materials and Methods section. (C) 0.5 × 10^6^/ml WEHI-231 cells were treated with 5 μM PP2 or an equivalent amount of DMSO for 48 hrs. Then the cells were spun down and stained with PI and AnnexinV-FITC for apoptosis analysis as described in the Materials and Methods section. (D) BKS-2 cells were treated with or without the indicated amount of PP1 (also a SFK inhibitor) or PP2 for 24 hrs. The cell lysates were probed for cyclin D2, then stripped and re-probed for β-actin. The ODs for cyclin D2 were normalized to the corresponding bands for β-actin and depicted as a fold change over no inhibitor.

Treatment with the SFK inhibitors PP2 or dasatinib induced predominantly G_1 _arrest in both BKS-2 and SudHL-4 cell lines in comparison to cells treated with the inactive analogue PP3 (Fig. 2A &2B) or the vehicle (data not shown), suggesting that SFK activity is needed for lymphoma cells to progress from G_1 _to S phase. PP2 had a similar effect on the proportion of cells in S phase in WEHI-231 (from 52.8% to 13.7%) and SUDHL-6 (from 46% to 23.4%) cells (data not shown). Since constitutive BCR signaling is also required for B lymphoma cell progression from G_1 _to S phase [3] and Igα and Igβ are thought to be the direct targets of Src kinase Lyn, the data are consistent with a role for constitutive Lyn activity in mediating constitutive B cell signaling to promote lymphoma growth. SFK inhibition also caused a modest increase in sub-G_1 _cells, indicative of apoptosis. To further confirm the effect of SFK inhibitors on apoptosis, WEHI-231 cells were treated with or without 5 μM PP2 for two days, which increased the apoptotic cells from 8% to 22% (Fig. 2C). PP2 and dasatinib also caused an increase in apoptosis in SudHL-4 cells (data not shown). These data collectively suggested that blocking SFK activity caused G_1 _- S arrest accompanied by apoptosis in B lymphoma cells.

The active complex of cyclin D/CDK4 targets the retinoblastoma protein for phosphorylation, allowing the release of E2F transcription factors to activate G_1_/S-phase gene expression. Since blocking SFK caused G_1_- S arrest for B lymphoma cells, we asked whether the level of cyclin D2 is affected by SFK inhibition. Treatment of BKS-2 with 10 μM PP2 (G1-S arrest shown in Figure 2B) for 24 hrs significantly reduced the protein level of cyclin D2 (Fig. 2D), consistent with SFK inhibition caused G_1 _- S arrest.

### Blocking SFK activity inhibits BCR proximal signaling events

**Figure 3:**
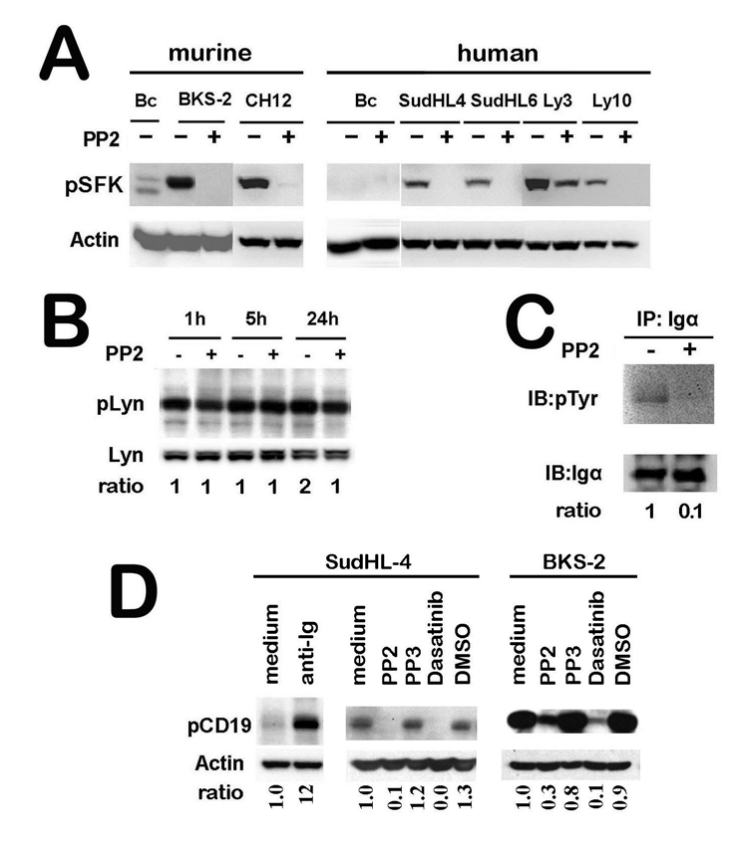
**PP2 inhibits the phosphorylation of Tyr396 in the activation loop of the kinase domain of Lyn or equivalent position in other SFKs, leading to a disruption of early BCR signaling events**. (Panel A) A number of B lymphoma cells were treated with 10 μM PP2 for 1 hr and then cell lysates were prepared for Western blotting. The blot was first probed with p-Src (Y416) antibody, then stripped and re-probed for β-actin. 'Bc' stands for primary B cells. (B) SudHL-4 lymphoma cells were treated with or without 10 μM of PP2 for the indicated time points and cell lysates were prepared for Western blotting. The blot was first probed with p-Lyn (Y507) antibody, then stripped and re-probed for total Lyn. The ODs for p-Lyn were normalized to total Lyn and depicted as a fold change over 1 hr without PP2. (C) SFK inhibition blocks constitutive phosphorylation of Igα in B lymphoma cells. SudHL-4 cells were treated with or without 10 μM PP2 for 1 hr. Igα was immunoprecipitated from equal amount of cell lysates from both groups and analyzed by Western blotting for p-Tyr. The lysates were also probed for Igα as a loading control. The ODs for p-Tyr were normalized to Igα and depicted as a fold change over no PP2 added. (D) SFK inhibition blocks constitutive phosphorylation of CD19 in B lymphoma cells. BKS-2 and SudHL-4 cells were treated as indicated for 4 hr. For a positive control, SudHL-4 cells were treated with 20 μg/ml anti-Ig for 15 min. The cell lysates were analyzed by Western blotting with pCD19 antibody. Then the blots were stripped and re-probed with anti-β-actin antibody. The ODs for pCD19 were normalized to the corresponding β-actin bands and then for each time point, depicted as a fold change over medium only for each panel.

Phosphorylation of SFK at the activation loop tyrosine was completely blocked upon treatment with 10 μM PP2 for all the cell lines tested except OCI-Ly3, which was reduced 50% but not completely eliminated (Fig. 3A). At a lower dose of PP1 or PP2 (1 μM), SFK phosphorylation is only slightly reduced (data not shown). As a control, phosphorylation of the carboxy-terminal Tyr507 of Lyn was not inhibited by 10 μM PP2 in SudHL-4 cells (Fig. 3B) and WEHI-231 cells (data not shown). This suggested that PP2 only inhibits phosphorylation of the tyrosine at the activation loop but not phosphorylation of the C-terminal inhibitory tyrosine in SFKs.

In normal B cells, the Src kinase, Lyn phosphorylates Igα and Igβ to mediate the BCR signaling pathway for B cell proliferation and differentiation. We hypothesized that Lyn is deregulated in B lymphoma cells and constitutively activates BCR signaling pathway to promote B lymphoma growth. To test that BCR is a direct target of Lyn, Igα was immunoprecipitated from SudHL-4 cell lysates treated with or without PP2 and then probed for p-Tyr. Phosphorylation of Igα was abrogated upon inhibition of SFK activity (Fig. 3C); consistent with the notion that Igα is a downstream target of Lyn. Since Lyn also activates PI3 kinase/AKT pathway by phosphorylating CD19, we asked whether phosphorylation of CD19 is inhibited upon blocking SFK activity. CD19 was constitutively phosphorylated in SudHL-4 and BKS-2 cells and was greatly enhanced by anti-Ig stimulation (anti-Ig data for BKS-2 is not shown). However, constitutive CD19 phosphorylation was blocked upon treatment with PP2 but not PP3 or vehicle.

### Phosphorylation of AKT and ERK, but not JNK is blocked upon SFK inhibition

**Figure 4:**
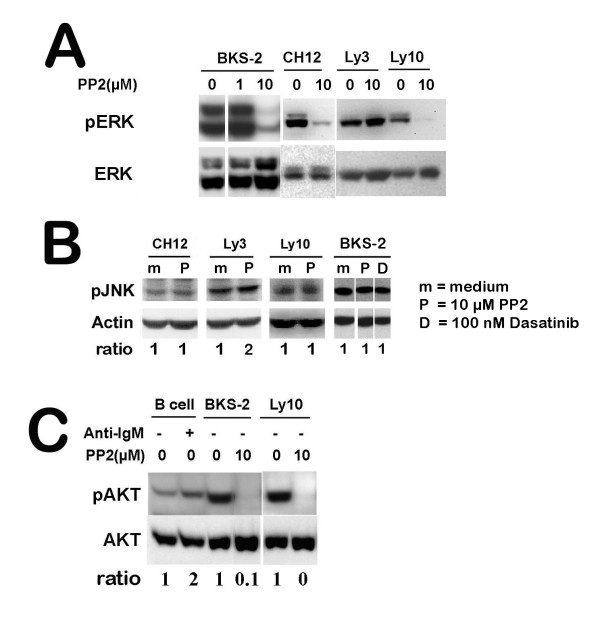
**SFK inhibition blocks the phosphorylation of ERK and AKT**. (Panel A) Four B lymphoma cells were treated with or without the indicated amount of PP2 for 1 hr. The cell lysates were analyzed by Western blotting for p-ERK. The blot was stripped and re-probed for total ERK. (B) Four B lymphoma cells lines were treated with or without PP2 for 1 hr. The cell lysates were analyzed by Western blotting for p-JNK. The blots were stripped and re-probed for β-actin. The ODs for p-JNK were normalized to the corresponding β-actin bands. For each cell line, the ODs are depicted as a fold change over no PP2. (C) BKS-2 and Ly10 lymphoma cells were treated with or without the indicated amount of PP1 or PP2 for 1 hr. As a positive control, normal splenic B cells were treated with 20 μg/ml anti-IgM for 15 min. The cell lysates were analyzed by Western blotting for p-AKT. The blot was stripped and re-probed for total AKT. The ODs for p-AKT were normalized to the corresponding bands for total AKT. For each cell type, the ODs are depicted as a fold change over no anti-IgM or PP2.

Since the early BCR signaling events are inhibited upon SFK inhibition, we next examined whether the further downstream pathways are affected as well. In B cells, ERK is a major downstream target that is phosphorylated in response to BCR signaling [37,49,50]. In BKS-2, CH12.Lx, OCI-Ly3, OCI-Ly10 lymphoma cells, we observed constitutive ERK activation (Fig. 4A), consistent with constitutively active BCR signaling. Treatment with 10 μM PP2 for 1 hr completely blocked the ERK phosphorylation in these lymphoma cells except OCI-Ly3, which requires higher dose of PP2 for complete blocking of SFK activity. At 1 μM PP1, which is not sufficient for blocking all the SFK activity, phosphorylation of ERK is not inhibited. Consistent with this, the proliferation of BKS-2 cells is not inhibited at this dose (data not shown).

Since ERK MAPK pathway is controlled by Src kinases, next we asked whether JNK MAPK is also controlled by Src kinases. PP2 (10 μM) does not affect the phosphorylation of JNK in CH12, Ly3, BKS-2, and Ly10 (Fig. 4B) and two other B lymphoma cell lines tested (data not shown), suggesting that JNK pathway is not controlled by Src kinases. Dasatinib as well did not reduce JNK phosphorylation in BKS-2 cells.

PI-3 kinase/AKT pathway is an important survival pathway activated in various cancer cells. In B cells, Lyn phosphorylates CD19 to activate PI-3 kinase/AKT pathway in response to antigen stimulation. Normal splenic B cells had very low levels of basal AKT phosphorylation which was enhanced by anti-IgM stimulation (Fig. 4C). In contrast, B lymphoma cells have higher levels of AKT phosphorylation and treatment with 10 μM PP2 completely blocked its phosphorylation (Fig. 4C). At a lower dose of PP2 (1 μM), the AKT phosphorylation is only slightly inhibited due to insufficient blocking of SFK activity (data not shown).

### Dasatinib inhibits the phosphorylation of SFK and its downstream targets

**Figure 5:**
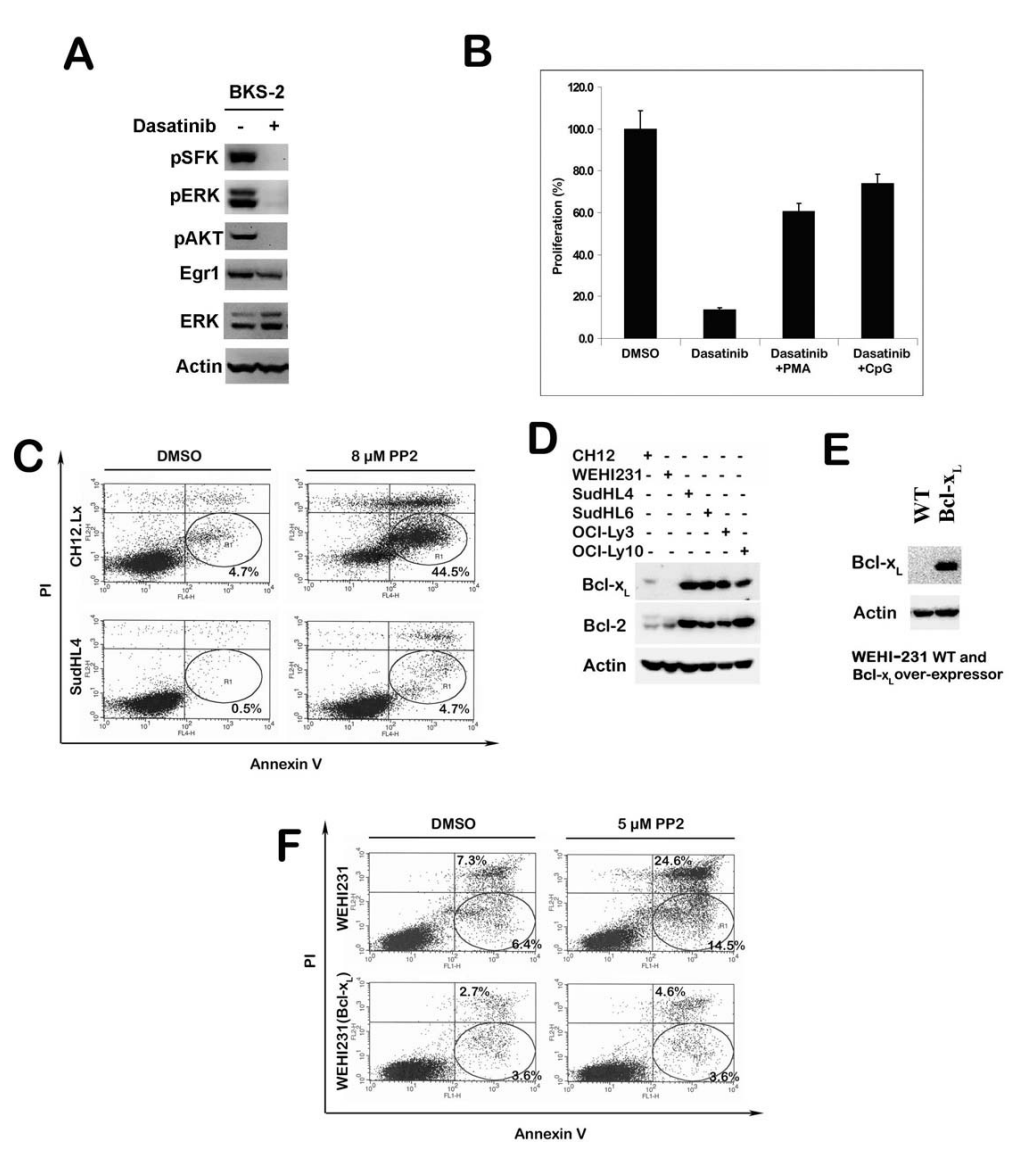
**Dasatinib inhibits constitutive BCR signaling and B lymphoma growth. Bcl-2 and Bcl-x_L _expression confers resistance to PP2 induced apoptosis in B cell lymphomas**. (A) Dasatinib inhibits SFK phosphorylation and phosphorylation of its downstream targets ERK and AKT and reduces the level of Egr-1 in BKS-2 lymphoma cells. BKS-2 cells were treated with or without 100 nM dasatinib for 1 hr. The cell lysates were analyzed by Western blotting for p-SFK, p-ERK and p-AKT. The blots were stripped and re-probed for Egr-1, ERK and β-actin. (B) Dasatinib induced growth inhibition can be partially overcome by PMA or CpG. BKS-2 cells were treated with DMSO, 10 nM dasatinib, 10 nM dasatinib+10 ng/ml PMA or 10 nM dasatinib+1 μg/ml CpG for 48 hrs. The proliferation assay was performed as described in the "Materials and Methods" section. Data points indicate percent control response ± S.E. of triplicate cultures from a representative experiment of three independent experiments. The percent control response is defined as (cpm in dasatinib treated group/cpm in DMSO treated group) × 100 for dasatinib; (cpm in dasatinib+PMA treated group/cpm in the PMA treated group) × 100 for dasatinib+PMA; (cpm in dasatinib+CpG treated group/cpm in CpG treated group) × 100 for dasatinib+CpG. The actual counts for DMSO, PMA and CpG treated groups are 16355 ± 1394, 12939 ± 1345, 89504 ± 9159, respectively. (C) SudHL-4 and CH12.Lx cells were treated with or without 8 μM PP2 for two days. Early apoptotic cells were detected by flow cytometry with AnnexinV-APC and PI staining as described in the "Materials and Methods". (D) Bcl-2 and Bcl-x_L _protein expression in six B lymphoma cells was determined. Cell lysates were analyzed by Western blotting for Bcl-x_L _and Bcl-2. The blots were stripped and re-probed for β-actin for loading control. (E) The Bcl-x_L _expression in WEHI-231 and WEHI-231-Bcl-x_L _cells (WEHI-231 stably transfected with Bcl-x_L_). Cell lysates were analyzed by Western blotting for Bcl-x_L_. Then the blot was stripped and re-probed for β-actin for loading control. (F) Ectopic expression of Bcl-x_L _makes WEHI-231 cells more resistant to PP2 induced apoptosis. WEHI-231 and WEHI-231-Bcl-x_L _cells were treated with or without 5 μM PP2 for one day. Early apoptotic cells were analyzed by flow cytometry with Annexin-V-FITC and PI staining as described.

Dasatinib was found to inhibit both BCR-Abl and Src kinases for Philadelphia chromosome-positive leukemia cells [51]. Since B lymphoma cells do not express BCR-Abl kinase, dasatinib is likely to inhibit the B lymphoma growth by blocking Src kinases. Treatment of BKS-2 cells with 100 nM dasatinib for 1 hr completely blocked the phosphorylation of SFK (Fig. 5A). As with PP1 or PP2, the phosphorylation of AKT and ERK was also completely blocked by dasatinib. In addition, the transcription factor Egr-1, which was shown by us to be critical for B lymphoma growth [35,37] was reduced 60% upon dasatinib treatment (Fig. 5A), probably due to the blocking of ERK activity [37].

Since Lyn is an early component of BCR signaling pathway, we next asked whether the effect of blocking SFK can be rescued by directly activating downstream pathways. Dasatinib (10 nM) potently inhibited the BKS-2 lymphoma growth by over 80%. The growth inhibition caused by dasatinib was partially rescued by PMA, an activator of PKC (60% rescue) or CpG ODN, an activator of MAPK and NF-κB (73% rescue) (Fig. 5B).

### Bcl-2 family proteins protect B lymphoma cells from SFK inhibition induced apoptosis

Although Lyn is important for B lymphoma growth, different B lymphoma cell lines exhibited different sensitivity to PP2 or dasatinib induced apoptosis. Notably the human diffuse large B cell lymphoma cell lines such as SudHL-4 were more resistant to PP2 induced apoptosis than murine cell lines such as CH12.Lx (Fig. 5C). Since Bcl-2 translocation is a hallmark for follicular lymphomas, we hypothesized that Bcl-2 family proteins have a role in protecting human diffuse large B cell lymphomas against SFK inhibition induced apoptosis. Accordingly we found that all the human diffuse large B cell lymphoma cells had abundant expression of Bcl-2 and Bcl-x_L _proteins compared to two murine immature B lymphoma cell lines (Fig. 5D). To further establish the correlation between Bcl-2 protein expression and resistance to SFK inhibitor induced apoptosis, WEHI-231 stably transfected with Bcl-x_L _was compared with parental WEHI-231 for PP2 induced apoptosis. Western blot showed that WEHI-231 stably transfected with Bcl-x_L _had abundant expression of Bcl-x_L _(Fig. 5E). PP2 induced less early apoptosis in WEHI-231- Bcl-x_L _cells than the parental WEHI-231 cells (Fig. 5F), suggesting that Bcl-2 family proteins protect B lymphoma cells from SFK inhibitor induced apoptosis.

### Lyn kinase is over expressed and constitutively active in B lymphoma cells

**Figure 6:**
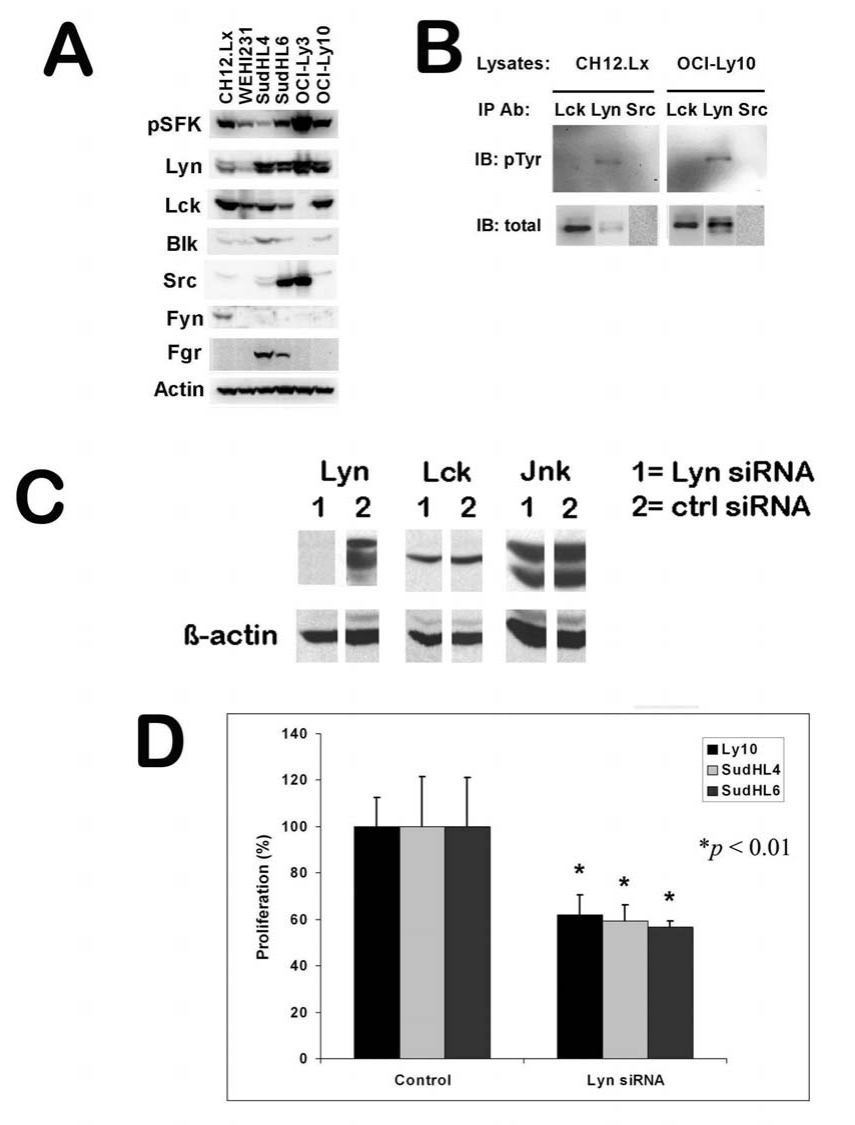
**Lyn is preferentially expressed, phosphorylated, and plays a critical role for B lymphoma growth**. (Panel A) Lyn and Lck are the predominant SFK expressed in a panel of six B lymphoma cell lines. Cell lysates from six representative B lymphoma cell lines were analyzed by Western blotting for p-SFK and different kinds of SFKs. The blots were stripped and re-probed for β-actin for loading control. (B) Lyn is constitutively phosphorylated in CH12.Lx and OCI-Ly10 lymphomas. Lck, Lyn or Src were immunoprecipitated from equal amount of cell lysates from CH12.Lx and OCI-Ly10 and probed for p-Tyr by Western blotting. The blots were then stripped and re-probed for total SFKs. (C) The Lyn siRNA suppressed Lyn expression in SudHL-4 lymphoma cells. SudHL-4 cells were transiently transfected with control or Lyn specific siRNA as described in the Materials and Methods section for 48 hrs. Multiple aliquots of cell lysates were run on SDS-PAGE and transferred onto PVDF membranes. The membranes were cut and separately probed for Lyn, Lck, and Jnk. The blots were stripped and re-probed for the loading control, β-actin. (D) Blocking Lyn expression by siRNA inhibited B lymphoma growth. OCI-Ly10, SudHL-4 and SudHL-6 lymphoma cell lines were treated with control or Lyn specific siRNA as described. Then an equal number of cells with the indicated treatment were used to set up the proliferation assay as described.

Since Src kinase activity is important for B lymphoma growth, we next asked which SFKs are preferentially expressed in B lymphoma cell lines. Most B lymphoma cell lines were found to predominantly express two to three SFKs (Fig. 6A). Lyn and Lck were abundantly expressed in four and five lymphoma cell lines, respectively. Two cell lines (SudHL-6 and OCI-Ly3) also expressed Src abundantly and Fgr was expressed in SudHL-4 and -6. Out of the panel of six SFKs tested, SudHL-4 expressed the largest number of PTKs. Yes and Hck were not detected in the six lymphoma cell lines tested (data not shown). To find which SFK is important for B lymphoma growth, we hypothesized that active kinases have phosphorylated tyrosine. As shown in Fig. 6B, Lyn is preferentially phosphorylated in both the lymphoma cell lines tested. In addition to Lyn phosphorylation, OCI-Ly3 also has constitutive phosphorylation of Src (data not shown). The data suggested that Lyn, in some cases plus Src, is the active SFK in B lymphoma cells.

To further evaluate the importance of Lyn for B lymphoma growth, Lyn specific siRNA was used to examine the effect of knocking down Lyn expression on B lymphoma growth. Western blot showed almost complete knock-down of Lyn expression in SudHL-4 cells (Fig. 6C). The expression of Lck protein, another member of the SFK family, as well as JNK, a MAPK, were unaffected by the Lyn siRNA treatment. Similarly phosphorylated as well as total Lyn levels were decreased in siRNA treated SudHL-6 cells (data not shown). Treatment of three lymphoma cell lines (including SudHL-4 for which the specific knock-down of Lyn by siRNA was shown in Fig. 6C) with Lyn specific siRNA caused a reduction of their growth by 40 - 50%. The reduction in growth is statistically significant.

### Blocking SFK activity inhibits the B lymphoma growth *in vivo*

**Figure 7:**
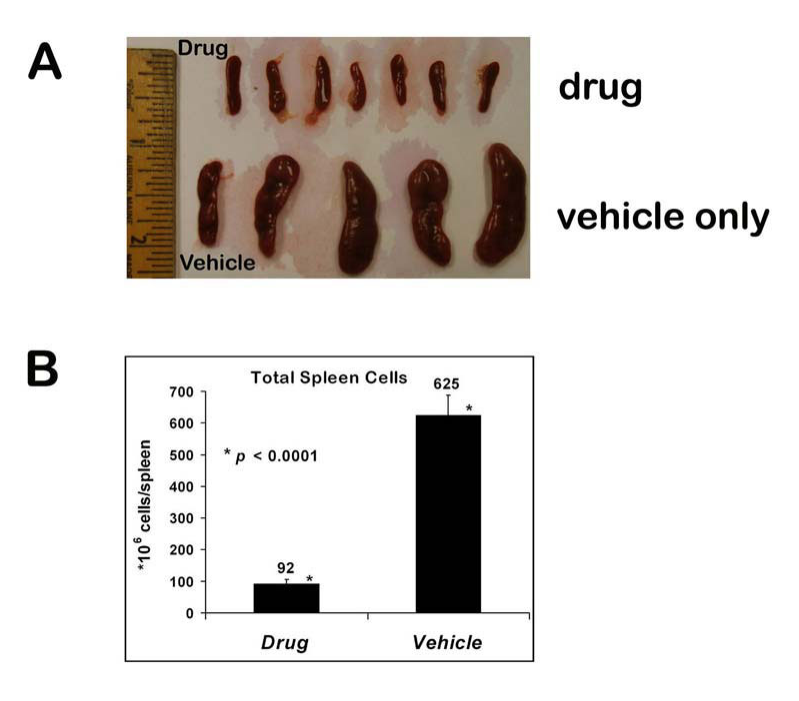
**Dasatinib inhibits B lymphoma growth *in vivo***. Dasatinib significantly reduced the splenic B lymphoma burden in CBA/N (Xid) mice. Female CBA/N (Xid) mice were injected intravenously with 7 × 10^6 ^BKS-2 B lymphoma cells on day 0. From day 1, mice were injected intraperitoneally either with drug (dasatinib) or vehicle (1 × PBS, 10% DMSO) everyday for 14 days. (A) A comparison of the spleen sizes between drug and vehicle treated lymphoma bearing mice. (B) The average counts of total splenic cells between drug and vehicle treated lymphoma bearing mice.

Since B lymphomas were susceptible to growth arrest upon treatment with dasatinib, we wanted to test if we could stop the growth of a B lymphoma in an *in vivo *lymphoma growth model. Twelve mice were divided into two groups and were injected with BKS-2 tumor cells. From the next day, seven mice got daily shots of dasatinib whereas the five control mice got only the vehicle. The seven dasatinib treated mice showed normal size of spleens (Fig. 7A, top row) whereas the five mice in the control group had greatly enlarged spleens due to expansion of tumor cells in the spleen (bottom row). The total number of cells in the spleen was increased from 92 × 10^6 ^per mouse for the drug treated group to 625 × 10^6 ^per mouse for the control group (Fig. 7B). Since a typical CBA/N (Xid) recipient mouse spleen has 50 × 10^6 ^cells, dasatinib treatment resulted in more than 13 fold reduction of tumor cells in the spleen.

## Discussion

According to the Leukemia & Lymphoma Society http://www.leukemia-lymphoma.org/hm_lls, as of 2009, an estimated 600,000 people are living with lymphoma in the U.S., most of which are NHLs (75%). Lymphoma incidence rose 79% from 1975 - 2005 and survival rates have not improved much in recent years. Identification of new drug targets will help improve treatment for lymphoma patients. Previously, our laboratory reported that constitutive BCR signaling is critical for B lymphoma growth [3]. We showed that expression of BCR co-receptors Ig-α and Ig-β and activation of the key downstream target Syk are critical for growth of established B lymphoma cells. As BCR signaling is dependent on SFKs, we investigated their role in B lymphoma growth in this study. We observed that Src kinase activity is constitutively elevated in a number of primary B lymphomas and diffuse large B lymphoma cell lines. Blocking Src kinase activity by specific pharmacological inhibitors (PP1, PP2 and dasatinib) inhibited the growth of these B lymphoma cells in a dose dependent manner. Dasatinib is an orally bioavailable drug that inhibits both BCR-ABL kinase and Lyn kinase. Dasatinib was shown to have better efficacy than Imatinib in treating BCR-ABL(+) CML [42]. In addition, dasatinib was shown to have activity against a variety of cancer cells including prostate cancer [52], lung cancers [53,54], head and neck squamous cell carcinoma [54], and human cancers associated with gain-of-function KIT mutations [41] etc. Here we report that dasatinib inhibits B lymphoma growth very potently with the IC_50 _in the nanomolar range (Table 1). Importantly, we also found that dasatinib strongly inhibited BKS-2 lymphoma growth *in vivo *in a mouse lymphoma model (Fig. 7), making it potential drug to be tested in combination with current therapies like R-CHOP [55].

When we examined six B lymphoma cell lines for protein expression of various SFKs, we found that Lyn and Lck are over expressed in five B lymphoma cell lines (Fig. 6A). Src is over expressed in two cell lines. It is a little surprising to see the expression of Lck in B lymphoma cells, although Lyn was more predominantly phosphorylated than Lck. It has now been shown that Lck is expressed in GC and mantle cell lymphomas [56,57] but rarely in non-GC B lymphomas [58]. The preferential phosphorylation of Lyn may be due to its association with BCR complex. Elevated expression and activity of Src have been reported in a variety of cancers [18,21]. Src was shown to be particularly important for tumor progression and metastasis [18]. We found that inhibition of OCI-Ly3 growth requires a much higher dose of inhibitors than any other lymphoma cell line tested (Table 1), probably due to over expression and phosphorylation of both Lyn and Src. Having both active Lyn and Src, this lymphoma may be a very aggressive tumor. The functional importance of Lyn was further confirmed since targeting Lyn with siRNA resulted in a ~50% reduction in proliferation for B lymphoma cells tested. A lack of more complete inhibition may relate to other Src kinases such as Lck or Src that may be able to substitute for Lyn's function after Lyn expression is knocked down. Nevertheless, since Lyn is predominantly expressed and constitutively phosphorylated in B lymphomas, Lyn activity probably accounts for the majority of the constitutive Src kinase activity seen for B lymphoma cells.

By cell cycle analysis, we found that blocking SFK activity induces G_1_-S growth arrest accompanied by apoptosis of B lymphomas. Consistent with this, we found reduced expression of cyclin D2 upon SFK inhibition (Fig. 2D). This is in agreement with a previous report that knocking down Igα by a siRNA approach caused G_1_-S growth arrest for B lymphoma cells [3], suggesting that blocking SFK activity inhibits constitutive BCR signaling to prevent cell cycle progression. Consistent with a predominant role of Lyn in B lymphoma cells, we observed that the BCR proximal signaling events were blocked upon inhibiting SFK activity, which includes blocking of the tyrosine phosphorylation for Igα and CD19. Furthermore, BCR downstream pathways such as phosphorylation of AKT and ERK, but not JNK were blocked upon SFK inhibition. Egr-1, a zinc finger transcription factor, shown to be important for B lymphoma growth [35] was also down regulated upon SFK inhibition. The data support an active role for Lyn kinase in mediating constitutive BCR signaling for lymphoma survival (CD19/PI3K/AKT pathway) and growth (ERK MAPK pathway). The SFK induced growth inhibition can be partially overcome by treating the cells with PMA or unmethylated CpG ODN. Since PMA directly activates the BCR downstream kinase, Protein Kinase C (PKC), hence ERK and Egr-1 [37], this suggests that the active PKC-ERK pathway can partially circumvent the blocking of BCR signaling caused by SFK inhibition. CpG activates Toll like receptor 9 mediated signaling pathways. CpG can rescue immature B lymphoma cells from BCR-mediated apoptosis by inducing a sustained activation of NF-κB, and subsequent expression of Bcl-x_L _and c-Myc [59,60] and an up regulation of Egr-1 [61].

In general, the human B lymphoma cell lines required higher doses of SFK inhibitors than murine B lymphoma cells to induce growth inhibition. There was very little apoptosis in the SFK inhibitor treated human B lymphomas. We showed that this could be related to increased expression of anti-apoptotic proteins Bcl-2 and Bcl-x_L _by the human B lymphomas compared to the murine lymphomas. Furthermore, constitutive expression of Bcl-x_L _made the WEHI-231 cell line less susceptible to SFK induced apoptosis.

Our data suggest that the constitutive BCR signaling in B lymphoma cells [3] is likely due to constitutive activation of Lyn, the upstream enzyme required for tyrosine phosphorylation of Igα and Igβ. Our studies are in general agreement with a recent report by Yang et al. [30] about the effects of dasatinib on lymphoma growth in vitro. They compared dasatinib to Imatinib to support the concept that SFK but not other tyrosine kinases are important for lymphoma growth. However, proteomic approaches have demonstrated that dasatinib can affect other PTKs like BTK, Csk, as well as other Ser/Thr kinases like p38α MAPK [62,63]. Therefore, our study used siRNA to specifically knock down Lyn and thus demonstrated Lyn is required for lymphoma growth. Furthermore, we were able to demonstrate dasatinib efficacy in an *in vivo *lymphoma model. The obvious question is: Why is Lyn kinase constitutively active in B lymphoma cells? One possibility is that Lyn is mutated in B lymphoma cells, which may be unlikely, since Lyn is active in a number of murine and human lymphoma cells. Another possibility is that Lyn is constitutively active due to the association of Lyn with lipid rafts that don't contain the negative regulator Csk in B lymphoma cells [64]. In normal B cells, Lyn is only transiently activated in response to BCR engagement by antigen. Singh et al showed that BCR engagement led to a Ca^2+ ^dependent, rapid production of reactive oxygen species (ROS), in particular H_2_O_2 _[65]. The ROS in turn led to a rapid and transient inhibition of protein tyrosine phosphatase (PTP) activity associated with the BCR due to the oxidation of the critical cysteine in the active site of PTP [66-69] and a transient increase in Lyn kinase activity [65]. Thus the extent of PTP oxidation determines the activation status of Lyn. In the light of this observation, and the data indicating a strong correlation between ROS and lymphomagenesis [68,69], it is conceivable that B lymphoma cells have a higher level of production of ROS than the normal B cells and the high level of ROS directly inactivates the PTPs, which causes phosphorylation and constitutive activation of Lyn. In support of this, we observed a higher level of global tyrosine phosphorylation in B lymphoma cells compared to the normal B cells (data not shown). It is interesting to note that phosphorylation on Tyr507 of Lyn did not keep Lyn inactive and Lyn is still phosphorylated on Tyr396. It may be that over expression of Lyn kinase promotes their aggregation and leads to autophosphorylation on Tyr396 first and an inactivation of SHP-1 by ROS keeps this phosphorylation stable. Once Lyn is phosphorylated on Tyr396, it may be less affected by the phosphorylation on Tyr507 due to an inactivation of CD45.

The complexity of the role of Lyn in B cells versus B lymphomas (negative and positive roles) is reminiscent of its negative role in normal myeloid cell development [11] and its positive role for the growth of chronic myeloid leukemia cells, where Lyn inhibitors are already being tested in clinic [70]. Similarly acute myeloid leukemia cells express constitutively active Lyn and their growth is inhibited by PP2 [71].

Overall, our studies suggest a model in which constitutive Lyn kinase activity phosphorylates Igα and Igβ to mediate the constitutive BCR signaling for B lymphoma survival and growth. Our data also suggest that like other types of cancers, B lymphomas are heterogeneous. In addition to having the constitutively active Lyn activity and constitutive BCR signaling, some lymphomas may have over expression of Bcl-2 anti-apoptotic proteins due to chromosomal translocation of BCL2 gene into the Ig loci. For those B lymphomas with Bcl-2 expression, small Src kinase inhibitors such as dasatinib in combination with Bcl-2 inhibitors such as ABT-737 [72] may be more effective than any single treatment.

## Abbreviations

BCR: B cell surface receptor; CML: chronic myelogenous leukemia; Csk: C-terminal kinase; DLBCL: Diffuse large B-cell lymphoma; ITAM: immunoreceptor tyrosine-based activation motifs; ITIM: immunoreceptor tyrosine-based inhibition motifs; NHL: Non-Hodgkin lymphoma; PBLs: human peripheral blood lymphocytes; PI: propidium iodide; PKC: Protein Kinase C; PTP: protein tyrosine phosphatase; ROS: reactive oxygen species; SFK: Src Family Protein Tyrosine Kinase.

## Competing interests

The authors declare that they have no competing interests.

## Authors' contributions

JK designed the study, performed experiments, and wrote the manuscript. RLC performed experiments and assisted in writing the manuscript. VS and DAR contributed to experiments. LL provided expertise with SFK inhibitors. CDJ provided primary human lymphoma samples. SB designed and supervised the study and manuscript preparation. All authors have read and approved the final manuscript.
